# Burtonian line in a patient with occupational lead poisoning

**DOI:** 10.1002/ccr3.5101

**Published:** 2021-11-19

**Authors:** Ali Banagozar Mohammadi, Mohammadreza Moslemi, Alireza Mirghaffari, Morteza Haramshahi

**Affiliations:** ^1^ Forensic Medicine and Medical Toxicology at Faculty of Medicine Tabriz University of Medical Sciences Tabriz Iran; ^2^ Department of Internal Medicine School of Medicine Tabriz University of Medical Sciences Tabriz Iran

**Keywords:** Burtonian line, Burton's line, lead line, lead poisoning, occupational poisoning

## Abstract

More attention of the medical staff to this sign can lead to faster diagnosis and timely treatment of patients.

## Graphical abstract

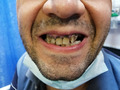

## 1 CASE PRESENTATION

In this article, we want to present a patient with Burtonian or lead line at the gum tooth line, a rare presentation of lead poisoning.

A 32‐year‐old man without any past medical history presented to the emergency department with a history of chronic abdominal pain. In addition, physical examination revealed bluish gingival pigmentation.

**FIGURE 1:**
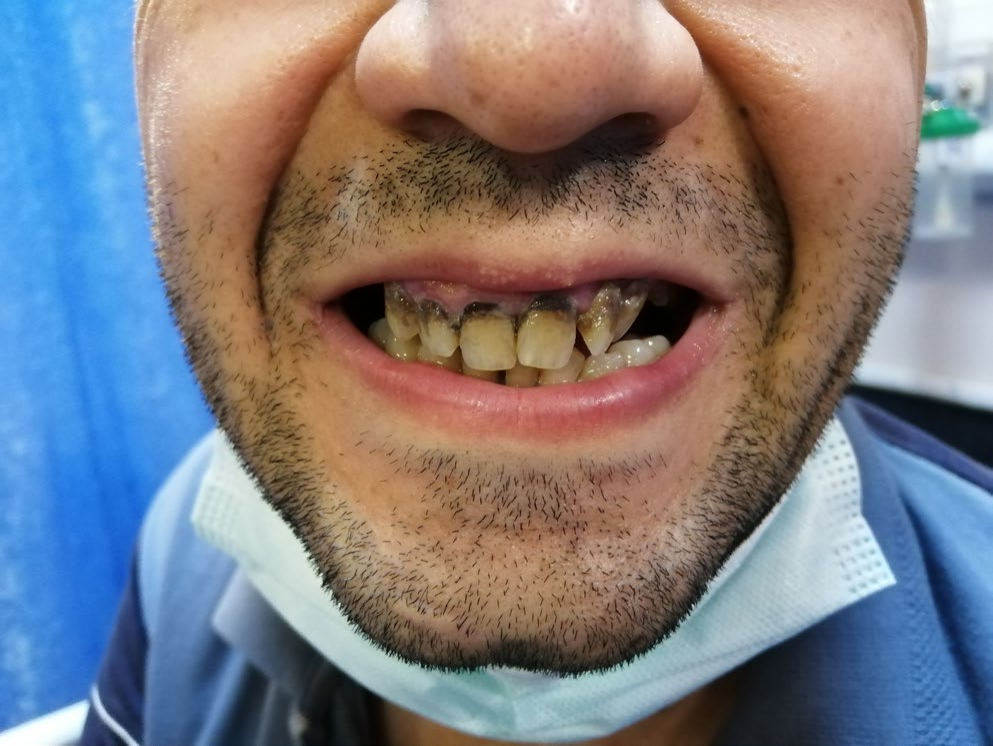
Bluish gingival pigmentation, also known as Burtonian or lead line, at the gum tooth line of the patient

The patient was a factory worker who had been in contact with molten lead in a battery factory for four years, so we decided to do related evaluations of lead poisoning for him. The laboratory testing revealed a lead blood level of 165 mg per deciliter (normal value, <25 mg per deciliter) and hypochromic microcytic anemia. Basophilic stippling was seen in the peripheral blood smear (Figure 1).

The patient has been admitted to our hospital's toxicology and poisoning department with the diagnosis of lead poisoning and has been treated with the succimer as a chelator therapy. Other treatments and diagnostic evaluations are being followed.

We consider bluish gingival pigmentation at the gum tooth line, Burtonian, or lead line, a rare presentation of lead poisoning and may not be presented if the patient had good oral hygiene.^1^

More attention to this rare sign can lead to faster diagnosis and timely discontinue of contact with the lead and prevention of chronic lead poisoning.

## CONFLICTS OF INTEREST

None.

## AUTHOR CONTRIBUTIONS

Mohammadreza Moslemi is the corresponding author and contributed to conception and design of the report and final approval of the version to be submitted. Ali Banagozar approved the final version of the submitted article. Alireza Mirghaffari prepared the figure and revised the article critically for important intellectual content. Morteza Haramshah drafted the article or revised it critically for important intellectual content.

## ETHICAL APPROVAL

This study was performed according to the principles outlined by the World Medical Association's Declaration of Helsinki on experimentation involving human subjects, as revised in 2000 and has been approved by the ethics committee of the Tabriz University of Medical Sciences.

## CONSENT

Written informed consent was obtained from the patient for the publication of this report and clinical images. Consent has been signed and collected in accordance with the journal's patient consent policy.

## Data Availability

The data that support the findings of the study are available from the corresponding author up on reasonable request.
